# Supplementary material for: The Construction and Exploration of a Comprehensive MicroRNA Centered Regulatory Network in Foxtail Millet (Setaria italica L.)
Source: Front Plant Sci. 2022 May 6;13:848474. doi: 10.3389/fpls.2022.848474 (PMC9121102; doi:10.3389/fpls.2022.848474)
Supplement: Supplementary file 1 [file Data_Sheet_1.zip › Sit-miR396c_Seita.3G361100.1_2996_TPlot.pdf]

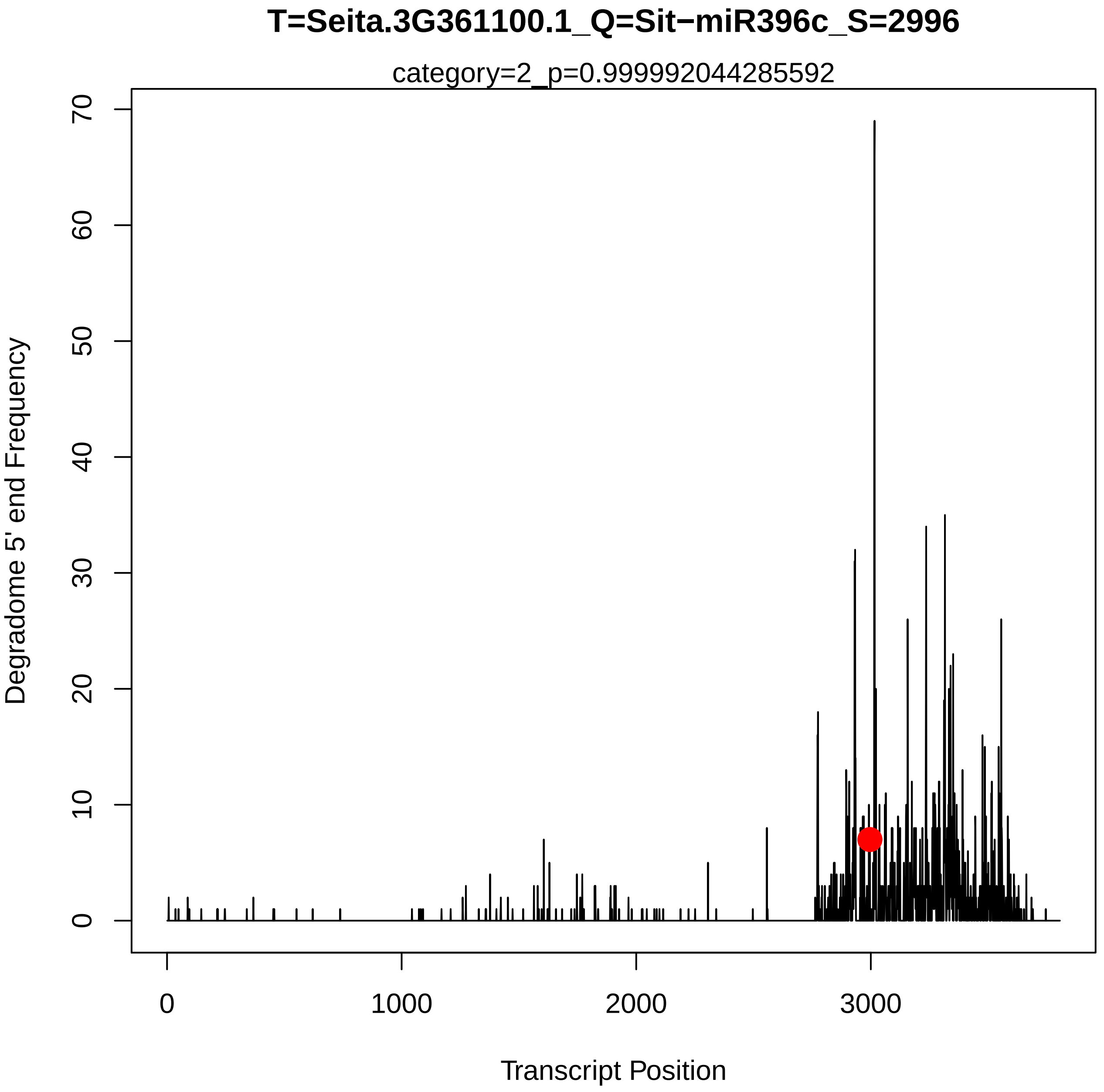

T=Seita.3G361100.1_Q=Sit−miR396c_S=2996
category=2_p=0.999992044285592
Degradome 5' end Frequency
0
10
20
30
40
50
60
70
0
1000
2000
3000
Transcript Position
